# Ambulatory Function and Perception of Confidence in Persons with Stroke with a Custom-Made Hinged versus a Standard Ankle Foot Orthosis

**DOI:** 10.1155/2012/206495

**Published:** 2012-05-17

**Authors:** Angélique Slijper, Anna Danielsson, Carin Willén

**Affiliations:** ^1^Department of Occupational Therapy and Physiotherapy, Skaraborg Hospital, 54185 Skövde, Sweden; ^2^The Institute of Neuroscience and Physiology, Sahlgrenska Academy at the University of Gothenburg, 41345 Gothenburg, Sweden

## Abstract

*Objective*. The aim was to compare walking with an individually designed dynamic hinged ankle foot orthosis (DAFO) and a standard carbon composite ankle foot orthosis (C-AFO). *Methods*. Twelve participants, mean age 56 years (range 26–72), with hemiparesis due to stroke were included in the study. During the six-minute walk test (6MW), walking velocity, the Physiological Cost Index (PCI), and the degree of experienced exertion were measured with a DAFO and C-AFO, respectively, followed by a Stairs Test velocity and perceived confidence was rated. *Results*. The mean differences in favor for the DAFO were in 6MW 24.3 m (95% confidence interval [CI] 4.90, 43.76), PCI −0.09 beats/m (95% CI −0.27, 0.95), velocity 0.04 m/s (95% CI −0.01, 0.097), and in the Stairs Test −11.8 s (95% CI −19.05, −4.48). All participants except one perceived the degree of experienced exertion lower and felt more confident when walking with the DAFO. *Conclusions*. Wearing a DAFO resulted in longer walking distance and faster stair climbing compared to walking with a C-AFO. Eleven of twelve participants felt more confident with the DAFO, which may be more important than speed and distance and the most important reason for prescribing an AFO.

## 1. Introduction

Hemiparesis is one of the most common impairments after stroke that contributes to reduced gait performance. The ability to walk is a primary goal for people with stroke and most stroke survivors regain the ability to walk [1]. Hemiplegic gait is characterized by decreased walking speed [2, 3] and energy inefficiency [2, 4]. Persons with hemiparesis walk significantly slower than healthy persons and after 6 months reach only 40–50% of the distance of age-matched healthy persons [5, 6]. As an adjunct to therapy, ankle-foot orthoses (AFO) are frequently used, although evidence is limited that an AFO improves elements of gait [7–11]. In one study, 22% of the stroke patients at a rehabilitation unit were discharged with an AFO [12]. An AFO can increase walking speed [8, 10, 13–15], improve walking stairs [8, 12], possibly decrease energy cost [14–16], and can be applied to partially correct the gait pattern [17, 18]. The swing phase of gait is especially facilitated by using an AFO, by compensating for excessive plantarflexion, lack of knee flexion, and toe extension [17, 18]. AFOs with support around the foot/ankle can improve the medio-lateral stability of the ankle during the standing phase [18]. In many hospitals in Sweden, when more stability in anterior/posterior direction around the ankle is needed, prefabricated carbon composite ankle foot orthoses (C-AFO) have frequently been chosen as a standard means. 

Several studies have been done on dynamic ankle foot orthoses (DAFO), which are AFOs individually designed with a custom-made footboard, support around the foot and dynamic parts such as hinges and springs [19–26]. The various studies have presented multiple variations of the DAFO, but all have in common the custom-made contoured footboard which is said to reduce spasticity [27]. DAFOs and C-AFOs are categorized as more rigid AFOs, prescribed to more severely affected patients, as they are meant to provide a relatively high degree of stability.

The effects of DAFOs and C-AFOs on more demanding locomotor tasks like climbing stairs, standing up from a chair, or walking a longer distance have not been studied.

Patients' experiences with and opinions about the use of the different AFO's have hardly been studied. Patients perspective is of importance for understanding compliance with use of prescribed aids [8, 11, 28, 29].

The aim of this study was to evaluate the effects of an individually designed DAFO compared to a standard C-AFO on different walking parameters, stair climbing, and perception of confidence in persons with chronic stroke.

## 2. Material and Methods

### 2.1. Subjects

Persons, who had been given a DAFO produced by the local Department of Prosthetics and Orthotics, were recruited from a rehabilitation clinic. Inclusion criteria were a stroke diagnosis with hemiparesis at least 6 months prior to the study, ability to walk for at least 6 minutes without personal assistance (walking aid was permitted), habituated to walking (at least one week) with a DAFO, and ability to walk with a C-AFO (habituated at least one week). Exclusion criteria were more than one stroke or gait other than stroke-induced disability, botulinum toxin treatment in either leg, pain while walking, or inability to follow instructions. 

Two men and 10 women (mean age 56 years, range 26–72) fulfilled the criteria and gave their informed consent. Median time since stroke onset was 25 months (range 7–312) (Table 1).

The study was approved by the Regional Ethical Review Board in Gothenburg.

### 2.2. Orthoses

**Figure 1:**
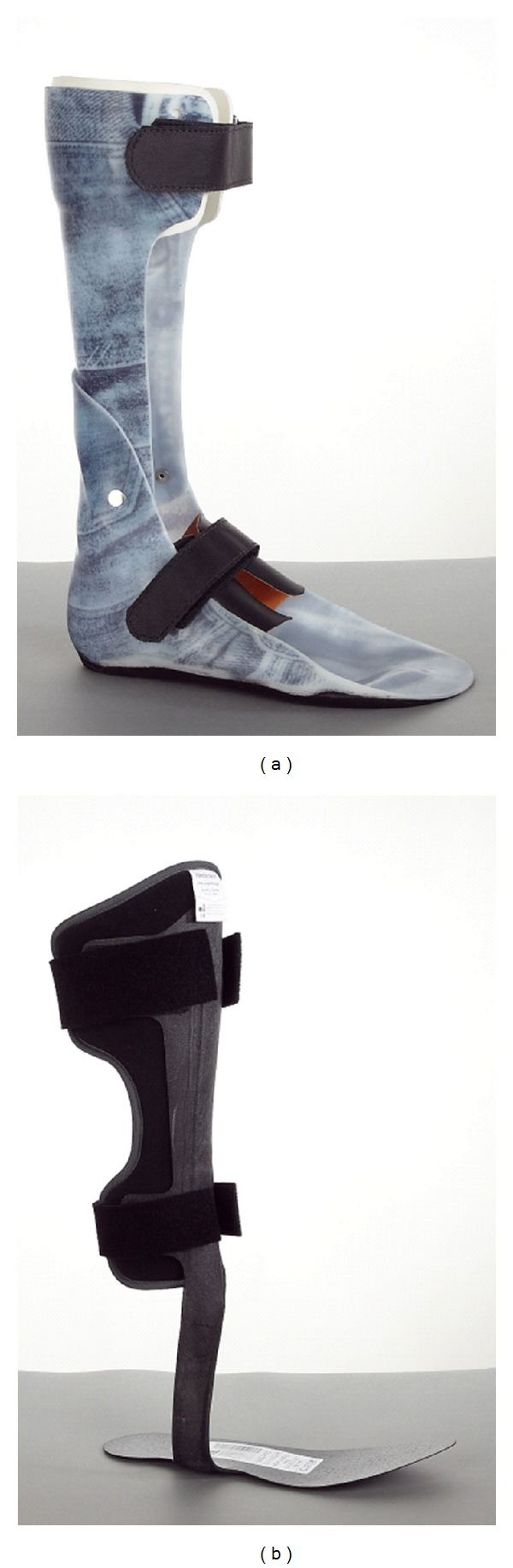
(a) Individually designed hinged ankle foot orthoses (DAFO), (b) Carbon composite ankle foot orthoses (C-AFO).

The individually designed hinged DAFO was a custom-molded, polypropylene, articulated AFO with a 90° plantar flexion stop and free dorsiflexion, manufactured by the local Department of Prosthetics and Orthotics. A custom-contoured full-length footplate was used to provide support to the natural arch contours of the foot. The impression for the DAFO was taken with the ankle in a neutral position, that is, with the tibia and foot aligned at a 90° angle (Figure 1(a)).

The C-AFO was an individually adjusted, standard carbon composite AFO (ToeOFF, Camp Scandinavia AB, Helsingborg, Sweden). The C-AFO consists of thermosetting matrix based on epoxy compounds reinforced with glass fiber, carbon, and Kevlar; it is claimed to have a dynamic component by absorbing energy at heel strike and returning it at toe-off (Figure 1(b)).

### 2.3. Measurement Methods

Dynamic gait analysis was performed using a telemetric foot pressure measuring system (T&T medilogic Medizintechnik GmbH, Mittelstrasse 9, D-12529 Schönefeldt, Germany). Key components of the gait analysis system were pressure-measuring insoles (available in different sizes), a connective sensor adaptor for additional sensors, as well as modems for the test subjects and computers. The system measured the walking velocity.

The 6-minute walk test (6MW) measures the distance walked (in meters) during a period of six minutes. The participants were instructed to walk at self-selected speed on a 75-meter figure eight walkpath situated in a 41-meter long and 2.68-meter wide corridor.

Heart rate (HR) was measured during the 6-minute walk test using a telemetric heart rate monitor (Polar Electro Oy, Professorintie 5, FIN-90440 Kempele, Finland). The Physiological Cost Index (PCI) was calculated as PCI = [HR at work−HR at rest (beats/min)]/walking speed (m/min) [30]. The value obtained stated in beats/meter represents the extra heart beats needed for walking compared to resting. The HR at rest was calculated as the lowest value measured during five minutes, while the participant was sitting in a quiet condition.

The participants' perceived exertion was rated on a horizontal visual analogue scale (VAS) ranging from 0 to 10 with 0 indicating no effort and 10 indicating maximal effort [31].

The walking velocity was registered using the telemetric system on a 10-meter section embedded in the 75-meter walkpath. The location of the 10-meter distance was unknown to the subjects, thus eliminating any effects of acceleration and deceleration. A difference of 0.2 m/s in walking speed was defined as clinically relevant, following a study of de Wit et al. [8]. 

The Stairs Test is an extended version of the Timed up and go test (TUG) [8]. The participants were timed while they rose from a chair with armrests, walked 1.15 m, ascended a flight of 12 stairsteps (deep 27 cm, height 16 cm, width 1.08 m), walked 1.65 m, touched the wall, turned around, descended the stairs, walked back to the chair, and sat down. A clinically relevant difference with the Stairs Test was defined as 15 seconds following de Wit et al. [8].

After completing the Stairs Test, the participants were asked to rate their perception of confidence on a Borg Category Rating Scale with one-point increments from 0 to 11/* (*maximal), with 0 indicating no feeling of confidence at all and 11/* indicating highest possible, maximal feeling of confidence [32]. The question to the person was “How confident did you feel when walking with this orthosis?”

The participants were tested on 2 occasions, median 7 days apart, at the same time of the day. The same physiotherapist (AS) conducted all the tests. Each test occasion lasted approximately 1.5 hours. At the first occasion, the participants' motor function was scored using the Fugl-Meyer Sensorimotor Assessment [33]. The presence of spasticity in the paretic calf muscle was assessed with the Modified Ashworth Scale (MAS) [34].

All subjects were equipped with a DAFO prior to the study and had worn this orthosis for various lengths of time. On the first test occasion, the participants were tested first with the DAFO and then with the C-AFO. Subjects wore the C-AFO for 0.5 hour prior to the tests with this device. The participants had one week of habituation during which they were only allowed to use the C-AFO during normal activities of daily life; the DAFO was kept at the clinic. The tests were repeated on the second test occasion, this time with the C-AFO first and after 0.5 hour of habituation, with the DAFO. The participants were instructed to walk around during the 0.5 hour of habituation.

The order of the walking tests was the same for all participants on both occasions, starting with the 6MW and rating of perceived exertion, followed by the Stairs Test and rating the feeling of confidence. The subjects wore their own comfortable shoes. They were allowed to use a walking aid during the walking test and stairs Test, and to use the handrail of the stairs on one side. The first test occasion was used to have the subjects get used to the test procedure; the values from the second test occasion were used for the present analysis.

### 2.4. Data Analysis

Statistical analysis was performed using SPSS Version 17.0. Conventional formulae were used for calculations of means, medians and standard deviations. Differences between the two test conditions at the second testing occasion were analysed using the paired *t*-test for continuous data, (as data was normally distributed) and the Wilcoxon's signed rank test for ordinal data. The significance level was set to *P* < 0.05.

## 3. Results

**Figure 2:**
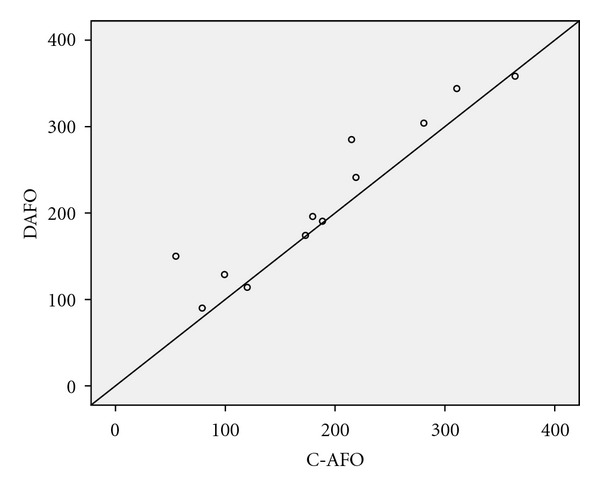
Individual walking distances (6MW) with DAFO and C-AFO, respectively.

The mean difference in walking distance (6MW) between the two orthoses in favor of the DAFO was 24.30 m (95% confidence interval (CI) 4.90 to 43.76) (Figure 2). The median (minimum, maximum) degree of experienced exertion rated after the 6MW was 3.3 (0.5, 8.0) when walking with the DAFO and 4.3 (1.0, 9.0) with the C-AFO. For the Stairs Test the mean difference was 11.80 s (−4.48, −19.05) in favor of the DAFO. No statistically significant difference was found between the two AFOs in either velocity for 10 m/walking or in PCI but in both instances the trend favored the DAFO. The median perceived confidence according to the Borg scale was 5.5 (3.0, 10.0) when walking with the DAFO and 1.5 (0.5, 5.0) with the C-AFO (*P*  value = 0.003) (Table 2). Analysis of data from the first testing occasion suggested very similar findings.

## 4. Discussion

The present study found that wearing an individually designed dynamic hinged ankle foot orthosis (DAFO) by hemiparetic persons resulted in increased walking distance and faster stair climbing, compared to walking with a standard carbon composite ankle foot orthosis (C-AFO). The median degree of experienced exertion and especially the degree of confidence were in favor of the DAFO. The study found almost no difference in velocity between the two orthoses, and the measure of energy cost also showed no statistically significant difference.

In a study of Bohannon [35], the participants considered walking independence to be very important followed by distance, compared to appearance and speed. In the present study, several participants were not able to walk at all without an AFO, which shows that the participants were severely affected by their hemiparesis. The total walking distance in this study was rather short so even if the mean difference of 24 meters in the present study may be not relevant in daily living, it can mean a big difference for some of the study participants. The six-minute walk test showed a statistical significant difference of approximately 13%, which has been described as the smallest real difference of clinical interest for the test [36]. Considering the short distance that the subjects walked in 6 minutes, it probably will take them a lot of time and energy to walking the longer distances needed in daily life. The fact that the three persons with the longest time passed after stroke and the most experience walking with a C-AFO still could increase their distance walked just by changing to a DAFO for one month, without any extra walking training, is of interest.

All participants except one perceived their degree of exertion to be lower when walking with the DAFO. The median difference was one cm on the 10 cm scale in favor of the DAFO. On the objective measurement of energy cost using the PCI, no difference between the two orthoses was shown. However, the mean PCI values of 1.08 and 1.17 with the DAFO and C-AFO, respectively, were very high compared to a value of 0.33 beats/m in healthy reference persons [37]. The PCI values in the present study are in line with other results on stroke subjects [38]. The differences in subjective and objective measures of energy cost may be explained by subjective preferences for the newer DAFO or by the fact that the PCI method may not be sensitive enough to detect a difference [39]. Several factors influence the energy cost of walking, and the relationships between them are complex.

In many studies, there is a focus on gait speed, which is considered to be of importance for independent walking. A change in gait speed of 0.20 m/s is said to be clinically relevant [8]. In the current study, the gait speed increased by 0.04 m/s when changing from the C-AFO to the DAFO, which was neither statistically nor clinically significant. In a study by Romkes, there was a difference of 0.03 m/s between two different orthoses, one of them a type of DAFO [20]. Some other studies comparing walking with and without AFO have found differences as large as 0.04, 0.045, and 0.07 m/s, which was statistically significant in two studies [8, 10, 28].

A feeling of confidence may be more important to persons with hemiplegia than speed and distance. Dogan reported that 35.3% of the study subjects who used an AFO indicated that they walked more confidently [40]. De Wit reported a 70% increase in self-confidence while using an AFO [8]. In the present study, 11 of 12 participants ranked their degree of confidence on an 11-point Borg scale a median of 3 scale steps higher for the DAFO compared to the C-AFO. The importance of this size of change is unknown, but it seems rather large. The use of different instruments to measure the perception of confidence/safety makes the studies difficult to compare to each other, however.

Interesting is to test tasks that challenge dynamic balance more than just walking on a level ground. Few studies report on stair walking ability. A Stairs Test detected improvement over time even in hemiparetic persons with a normal walking speed [3]. The Stairs Test results tell more about the functionality of ambulation than does walking velocity measured on a straight, level track. The current study showed a difference of 11.8 s between the two different orthoses in the Stairs Test, compared to 8.6 s in a study of de Wit whose comparison was between walking with and without AFO [8]. The stairs Test showed a statistical difference of approximately 21% which has been described as the smallest real difference of clinical interest for a similar test [36]. There were several participants with a clinically relevant difference on the Stairs Test in favor of the DAFO, like 25 s for one participant, 20 s for another, and 41 s for a third. Even here the participants with the longest time passed after stroke improved their result in the Stairs Test, just by changing from the C-AFO to the DAFO.

A limitation of this study is the small sample size. The time for habituation to the C-AFO varied and may have been too short for some of the participants for a fair comparison. However, half of the twelve subjects had used the C-AFO longer than the DAFO. The order of testing was not randomized at the second test occasion, which may have influenced the results. However, on the first occasion the test order had been the reverse (DAFO before C-AFO) with similar results. A strength with the study is that two measurement occasions were used, with the first trial serving as practice.

## 5. Conclusions

In subjects with hemiparesis, an individually designed dynamic ankle foot orthosis can be a better alternative than a standard ankle foot orthosis.

The person's level of confidence can be increased when walking with an individually designed dynamic foot orthosis compared to a standard AFO, which may be more important than speed and distance improvements.

## Figures and Tables

**Table 1:** Description/Characteristics of the study population.

Sex	Age (years)	Hemiparetic side	Time since stroke (months)	Motor function^a^	Muscle tone^b^	Time with DAFO (months)	Time with C-AFO (months)	Walking aid
F	26	Right	10	24	3	7.5	0.25	
F	51	Left	14	23	0	10	0.25	
F	63	Right	10	27	0	1	0.25	crutch
M	54	Right	312	20	3	2	26.5	
F	35	Right	36	32	0	6	24	
F	65	Left	12	22	3	8	0.25	cane
F	74	Left	55	12	2	3.5	48	cane
F	72	Left	65	25	2	1	64	cane
F	61	Right	200	29	3	1	51	pole
F	67	Right	10	16	3	8	0.25	crutch
M	56	Left	56	23	4	13.5	6	quadripod
F	52	Right	7	27	1	2.5	3	

^a^ FMA: a Fugl-Meyer Sensorimotor Assessment, leg section, maximum 34.

^b^ Modified Ashworth Scale, calf muscle, 0–5.

**Table 2:** Results of walking tests and differences between the two orthoses.

	DAFO	C-AFO	Difference	95% CI	*P* value
6MW (m)	214.7 (90.9)	190.3 (94.7)	24.3	4.90−43.76	0.019
PCI (beats/m)	1.08 (1.35)	1.17 (1.35)	−0.09	−0.27−0.95	0.320
Velocity (m/s)	0.59 (0.25)	0.55 (0.28)	0.04	−0.01−0.10	0.127
Stairs (s)	57.9 (32.1)	69.7 (42.6)	−11.8	−19.05–4.48	0.005
Perceived exertion VAS (0–10)	3.3 (0.5, 8)	4.3 (1, 9)	−3.3 (−9.5, 1)		0.038
Perceived confidence Borg CR scale (0–11)	5.5 (3, 10)	1.5 (0.5, 5)	−3 (−9, 1)		0.003

Values for 6MW, PCI, Velocity, and Stairs are given in mean and standard deviation. Medians (minimum and maximum observed values) are presented for Perceived exertion VAS and the Perceived confidence Borg CR scale.
